# Factors Contributing to Diagnostic Discordance Between Store-and-Forward Teledermatology Consultations and In-Person Visits: Case Series

**DOI:** 10.2196/24820

**Published:** 2021-02-25

**Authors:** Michelle S Lee, Robert Stavert

**Affiliations:** 1 Harvard Medical School Boston, MA United States; 2 Department of Dermatology Cambridge Health Alliance Somerville, MA United States

**Keywords:** teledermatology, eHealth, dermatology, telemedicine, diagnosis

## Abstract

**Background:**

Use of asynchronous store-and-forward (SAF) teledermatology can improve access to timely and cost-effective dermatologic care and has increased during the COVID-19 pandemic. Previous research has found high diagnostic concordance rates between SAF teledermatology and face-to-face clinical diagnosis, but to our knowledge, none have used specific cases to illustrate factors contributing to diagnostic discordance.

**Objective:**

To identify and illustrate characteristics that may have contributed to diagnostic discordance between store-and-forward teledermatology and in-person clinical diagnosis in a series of patients.

**Methods:**

We identified 7 cases of diagnostic discordance between teledermatology and in-person visits where the favored diagnosis of the in-person dermatologist was not included in the differential diagnosis formulated by the teledermatologist. Cases were identified from a previously published retrospective chart review of 340 SAF teledermatology consultations, which was previously performed at an academic community health care system in the greater Boston area, Massachusetts, from January 1, 2014, through December 31, 2017. Of 99 patients who completed an in-person dermatology appointment after their teledermatology consultation, 7 had diagnostic disagreement between the teledermatologist and in-person dermatologist where the diagnosis in the in-person consultation was not included in the differential diagnosis in the original teledermatology consult. These 7 cases were examined by 2 author reviewers to identify factors that may have contributed to diagnostic discordance.

**Results:**

Factors contributing to diagnostic discordance between SAF teledermatology consultations and in-person visits included poor image quality, inadequate history or diagnostic workup, inability to evaluate textural characteristics, diagnostic uncertainty due to atypical presentations, and evolution in appearance of skin conditions over time.

**Conclusions:**

We identified multiple factors that contributed to diagnostic discordance. Recognition and mitigation of these factors, when possible, may help to improve diagnostic accuracy and reduce the likelihood of misdiagnosis. Continuing education of referring providers and implementation of standardized guidelines for referrals may also be helpful in reducing the risk of misdiagnosis due to inherent limitations of teledermatology services.

## Introduction

Store-and-forward (SAF) teledermatology systems utilize asynchronous evaluation of clinical images and information to provide diagnostic and management guidance directly to patients or other health care providers. In contrast to real-time telemedicine such as video encounters, SAF encounters involve collecting clinical information from a referring provider to be sent electronically to another site or provider, often a specialist, for review at a later time. SAF teledermatology platforms can increase access to dermatologic care, provide financial savings for patients and health systems, and provide a comparable quality of care to in-person evaluation for numerous dermatologic conditions [1-8]. Utilization of both synchronous video and asynchronous SAF telemedicine has increased significantly during the 2020 COVID-19 pandemic [9,10]. SAF teledermatology may play a particularly vital role in the provision of safe and efficient dermatologic care as it requires less resources and coordination to implement compared to live interactive teledermatology [11].

Evaluation of diagnostic concordance for patients who receive both a teledermatology and in-person consultation is one method of assessing the diagnostic quality of SAF teledermatology consultations. Complete diagnostic concordance occurs when the first diagnosis matches between the in-person dermatologist and teledermatologist [12-17]. Previous research has found 79%-94% concordance rates between teledermatology and face-to-face clinical diagnosis, with some variation based on factors including skin condition and whether or not dermatoscopy is utilized [12-17]. High rates of diagnostic concordance help to ensure that the diagnoses patients receive from SAF teledermatology platforms are comparable to those that patients would receive during an in-person encounter.

Although previously published work has examined rates and patterns of discordance [12-17], to our knowledge, none have previously used cases to identify and illustrate specific characteristics that may contribute to diagnostic discordance. We analyzed a series of 7 cases of diagnostic discordance, identifying contributing factors in hopes of identifying opportunities to improve teledermatology systems and mitigate potential risks that can occur from misdiagnosis.

## Methods

Previously, a retrospective chart review of 340 SAF teledermatology consultations performed at our institution from January 1, 2014, through December 31, 2017, was conducted [18]. All SAF teledermatology cases were ordered alphabetically by patient’s last name, and the first 340 cases were reviewed. Among these 340 teledermatology cases, there were 99 patients who also completed an in-person dermatology visit, and further chart review was performed to determine the level of management concordance between teledermatologist and in-person dermatologist, defined by five categories: (1) fully concordant, (2) partially concordant, (3) discordant, (4) unable to assess because treatment was not specified by the referring provider, and (5) treatment not specified by teledermatology provider and an in-person appointment is requested for further evaluation. The definition of diagnostic discordance for this study was based on previous literature, which has defined diagnostic concordance as complete agreement (where the first diagnosis matched between in-person dermatologist and teledermatologist), partial agreement (where diagnoses overlapped between in-person dermatologist and teledermatologist), and discordant (where diagnoses did not match between teledermatologist and in-person dermatologist) [18-20]. Analysis of the 99 patients with both teledermatology and in-person visits found that diagnoses in 76 (77%) encounters were fully concordant, 16 (16%) were partially concordant, and 7 (7%) were fully discordant. We further evaluated these 7 diagnostically discordant cases to identify factors contributing to diagnostic discordance. Both authors (MSL and RS) performed retrospective chart review of the cases and discussed causes of the diagnostic discordance to come to a consensus.

Images from the teledermatology consult were submitted by the referring provider and taken using the Epic Haiku mobile app (Epic Systems Corporation). The teledermatologist was different than the in-person dermatologist in all but the second case reviewed. This project was exempt from full review by our Institutional Review Board.

## Results

### Overview

A summary of the cases, teledermatology and in-person differential diagnoses, in-person diagnosis, and factors contributing to diagnostic discordance is provided in Table 1.

**Table 1:** Summary of cases and factors contributing to diagnostic discordance.

Case	Age (yrs) and gender	Telederm^a^ differential diagnosis	Treatment after telederm visit	In-person differential diagnosis	In-person diagnosis (diagnostic test)	Contributing factors to diagnostic uncertainty
1	31, male	Superficial morphea, superficial dermatophyte infection, pityriasis rotunda, psoriasis, parapsoriasis, CTCL^b^, Hansen	None	Confluent and reticulated papillomatosis of Gougerot and Carteaud, morphea, tinea corporis, and discoid erythrasma	Eczematous dermatitis (punch biopsy)	Image quality compromised by patient positioning and lighting leading to shine artifact; unusual morphology, presentation; lack of historical details provided
2	60, male	Sarcoid, mycobacterial, hypersensitivity, lichenoid reaction, pseudolymphoma, arthropod bite, folliculitis	Betamethasone dipropionate 0.05% cream twice a day	Tinea corporis, sarcoid, annular lichen planus	Tinea corporis (KOH prep^c^ confirmed)	Image quality compromised by limited view of anatomic area; evolution of rash from time of telederm to time of in-person visit from papular to characteristic annular with scale
3	26, male	Impetigo, tinea faciei, Majocchi, contact dermatitis, rosacea	Empiric doxycycline 100 mg orally twice a day for 2 weeks, continue clotrimazole	VZV^d^ reactivation (herpes zoster)	Herpes zoster (physical exam)	Limited single-image view made it more difficult to appreciate dermatomal distribution; lack of adequate testing including superficial bacterial culture and KOH prep; evolution of rash from telederm consult to time of in-person visit
4	63, male	Actinic keratoses, excoriated papulopustular rosacea, squamous cell carcinoma	None prescribed	Telangiectasias due to sun damage	Nonspecific telangiectasias due to sun damage (clinical diagnosis)	Inability to palpate lesion to determine textural characteristics; image artifact showing overlying scale; no dermatoscopic images taken during initial consultation
5	69, male	HSV^e^, erythema multiforme, contact dermatitis, or pemphigus vulgaris, and paraneoplastic pemphigus	None prescribed	Allergic contact dermatitis vs actinic cheilitis	Lichenoid dermatitis (biopsy)	HSV/VZV viral culture would have been helpful when evaluating vesicles on mucosal surfaces; diagnostically challenging case; lack of historical details provided
6	41, male	HSV, LGV^f^	None prescribed	Condyloma acuminata	Epidermal inclusion cyst (biopsy)	Nonclassic presentation resulting in diagnostic uncertainty; viral and bacterial swab cultures would be helpful for initial consult; difficulty in distinguishing between vesicles, pustules, cysts via telederm
7	65, male	Unable to determine	None prescribed	Irritated seborrheic keratosis vs melanoma	BCC^g^ (shave biopsy)	Dermatoscopic images were out of focus, and nondermatoscopic images were not included

^a^Telederm: teledermatology.

^b^CTCL: cutaneous T-cell lymphoma.

^c^KOH prep: potassium hydroxide preparation.

^d^VZV: varicella zoster virus.

^e^HSV: herpes simplex virus.

^f^LGV: lymphogranuloma venereum.

^g^BCC: basal cell carcinoma.

### Case 1

**Figure 1:**
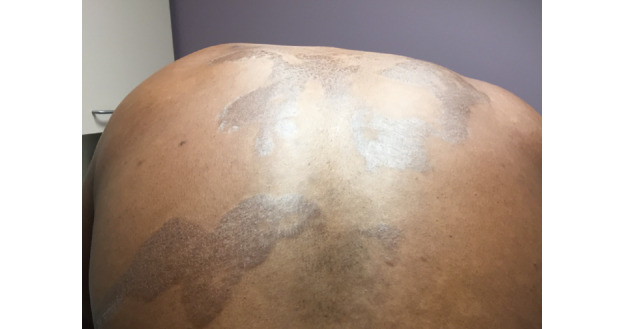
Case 1. Submitted teledermatology image showing patient’s back with hyperkeratotic plaque and xerotic scale. Image quality compromised by patient positioning and lighting leading to shine artifact.

**Figure 2:**
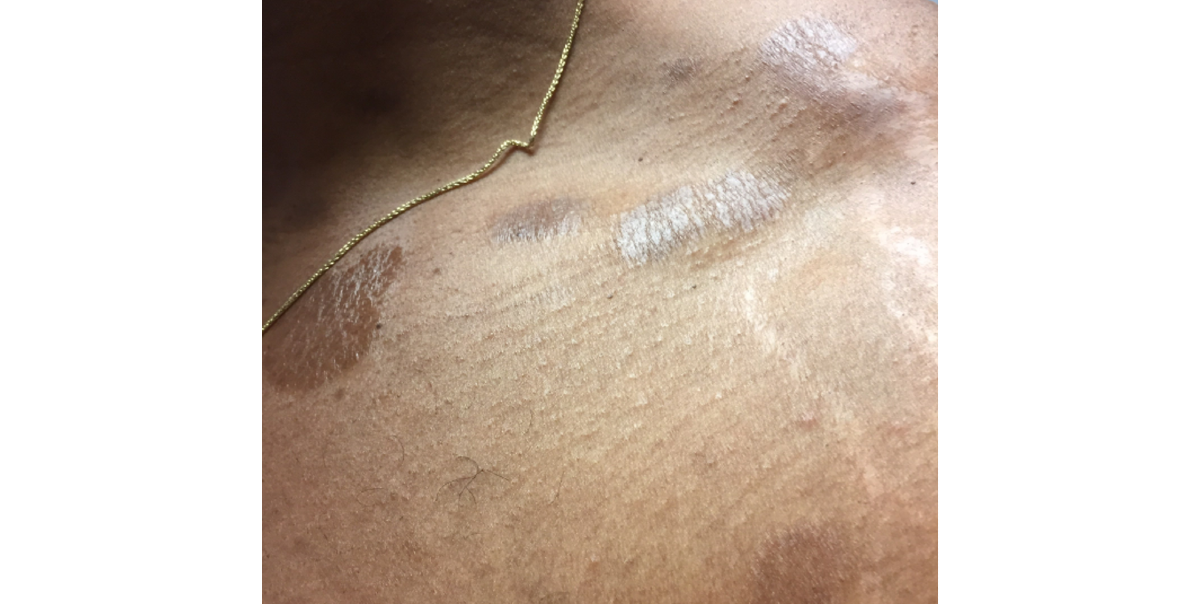
Case 1. Submitted teledermatology image showing patient’s neck and clavicular region with hyperkeratotic plaque and xerotic scale. Image quality compromised by patient positioning and lighting leading to shine artifact.

**Figure 3:**
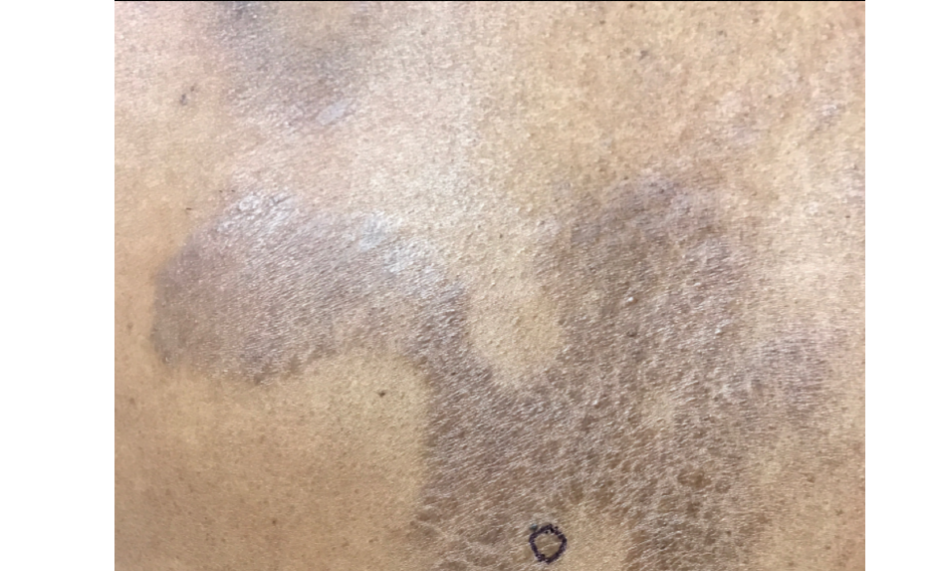
Case 1. Image for in-person visit showing well-demarcated, geographic, hyperpigmented, atrophic, and wrinkly patches on the back.

A 31-year-old male presented to his primary care provider with a several-year history of well-circumscribed, hyperpigmented, nonpruritic, thin, scaly plaques with skin tightening on his back, trunk, and chest, as well as associated gynecomastia. The patient had tried applying moisturizing lotion without relief. Teledermatology consultation resulted in a broad differential diagnosis including superficial morphea, superficial dermatophyte infection, pityriasis rotunda, psoriasis, parapsoriasis, cutaneous T-cell lymphoma, and Hansen disease. The submitted images showed several sharply demarcated hyperpigmented thin plaques with overlying xerotic scale on the back as well as well-circumscribed tan thin plaques with overlying scale on the collar distribution of the neck and the upper chest (Figures 1 and 2). The teledermatologist noted that the image quality was limited by patient positioning and lighting, leading to shine artifact, and noted that further history about potential exposures and travel history would have been helpful, particularly to rule out Hansen disease. Due to the broad differential and no leading diagnosis, he was referred for an in-person consultation. His in-person exam revealed well-demarcated geographic hyperpigmented atrophic and wrinkly patches on the back (Figure 3) and the anterior bilateral shoulders, left flank, and upper arms, as well as gynecomastia. The differential diagnosis included confluent and reticulated papillomatosis of Gougerot-Carteaud, morphea, tinea corporis, and discoid erythrasma. A punch biopsy was performed, which revealed findings most consistent with an eczematous dermatitis. The patient was treated with triamcinolone 0.1% cream and did not return for scheduled follow-up appointments.

### Case 2

**Figure 4:**
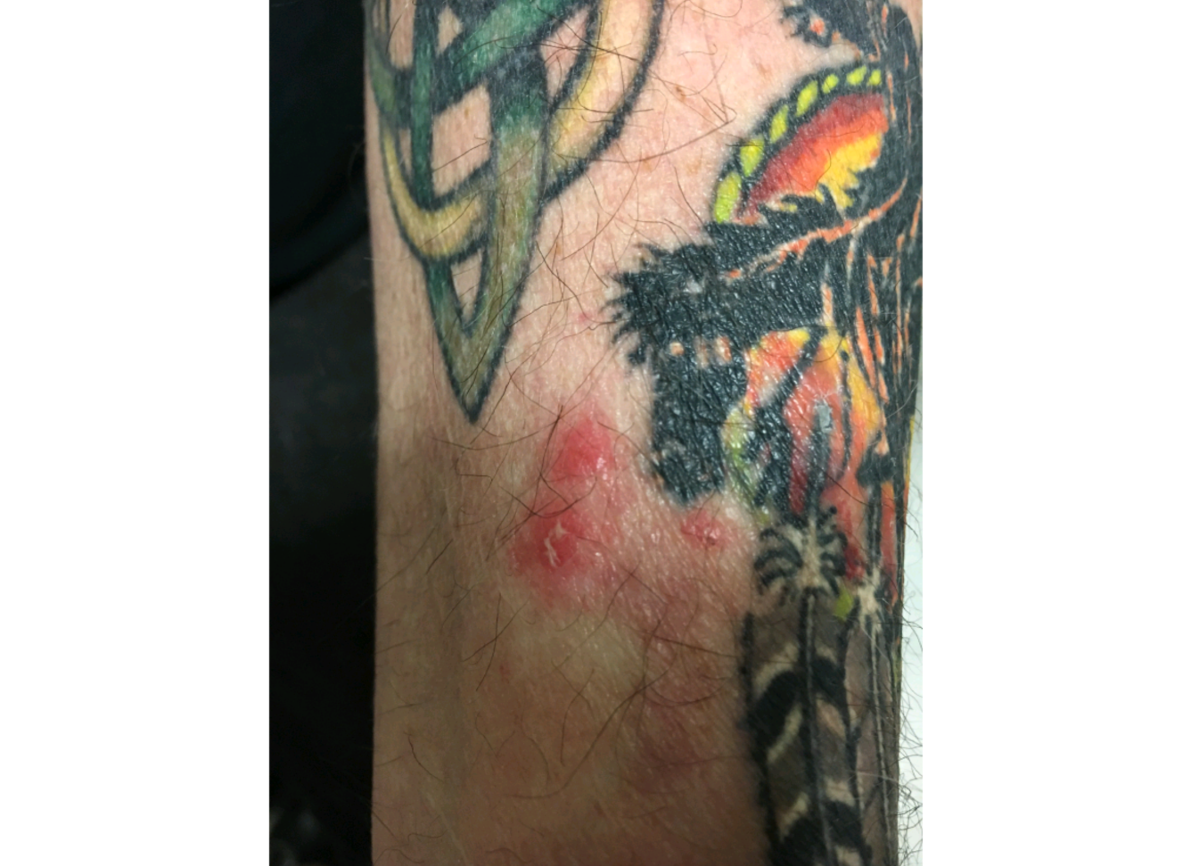
Case 2. Submitted teledermatology image showing left forearm with 1-2 cm light pink patch containing three discrete 4-6 mm pink papules. Image quality compromised by limited anatomic view.

**Figure 5:**
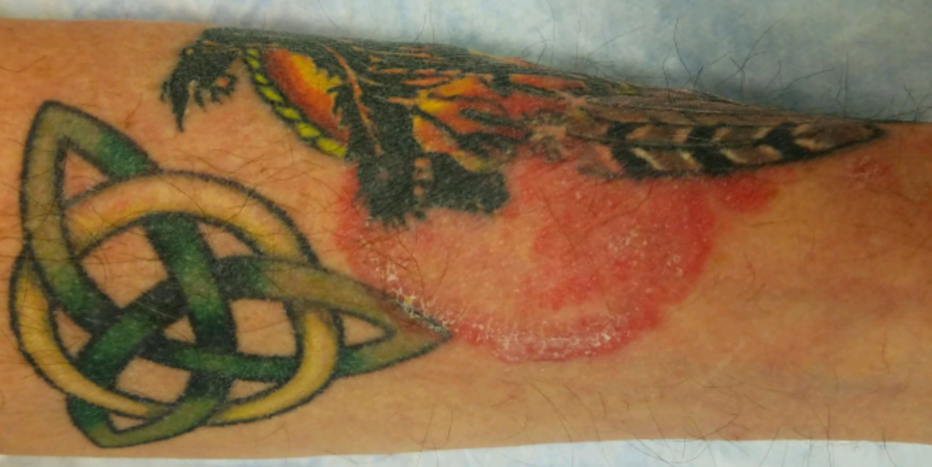
Case 2. In-office photo obtained from same patient, demonstrating left forearm with pink-red annular plaque with scale (tinea corporis).

A 60-year-old man with 3 weeks of pruritic pink papules on the left forearm next to tattooed skin was referred to teledermatology. Submitted photos showed a 1-2 cm light pink patch containing three discrete 4-6 mm pink papules, and the differential diagnosis included sarcoidosis, atypical mycobacterial infection, hypersensitivity reaction, lichenoid reaction, and pseudolymphoma, as well as arthropod assault and folliculitis. The teledermatologist noted that image quality was compromised by the limited anatomic view provided (Figure 4). The patient was prescribed betamethasone dipropionate 0.05% cream twice daily for 2 weeks. In the office 3 weeks later, he was noted to have a pink-red annular plaque with overlying scale (Figure 5) that was suspicious for tinea corporis, which was confirmed with a potassium hydroxide preparation (KOH prep) showing hyphae. The patient was treated with topical ketoconazole 1% cream, and his rash resolved without recurrence.

### Case 3

**Figure 6:**
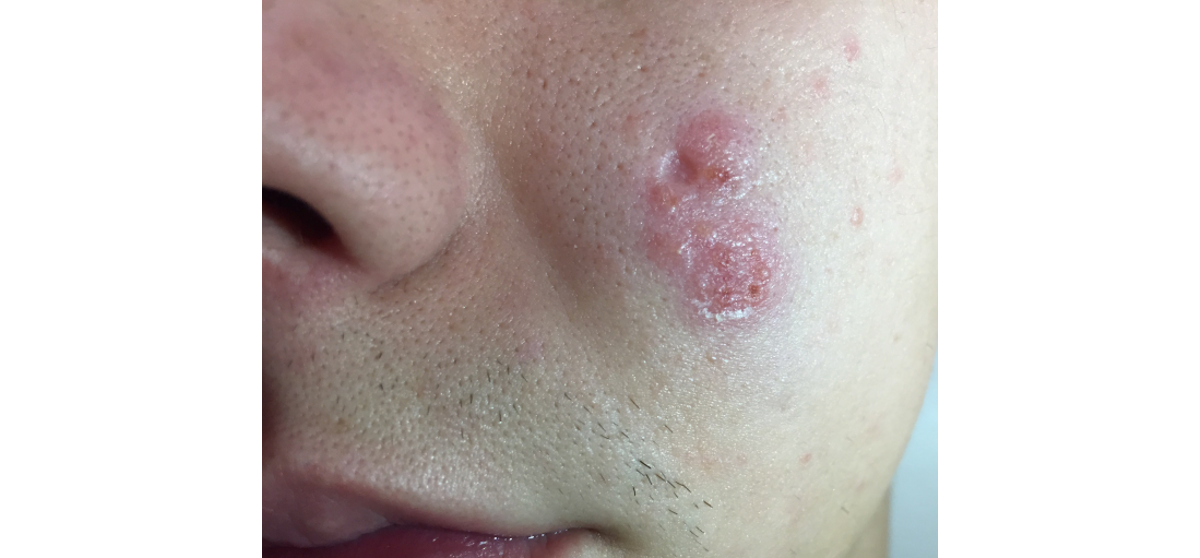
Case 3. Limited view of the face from teledermatologist consult.

**Figure 7:**
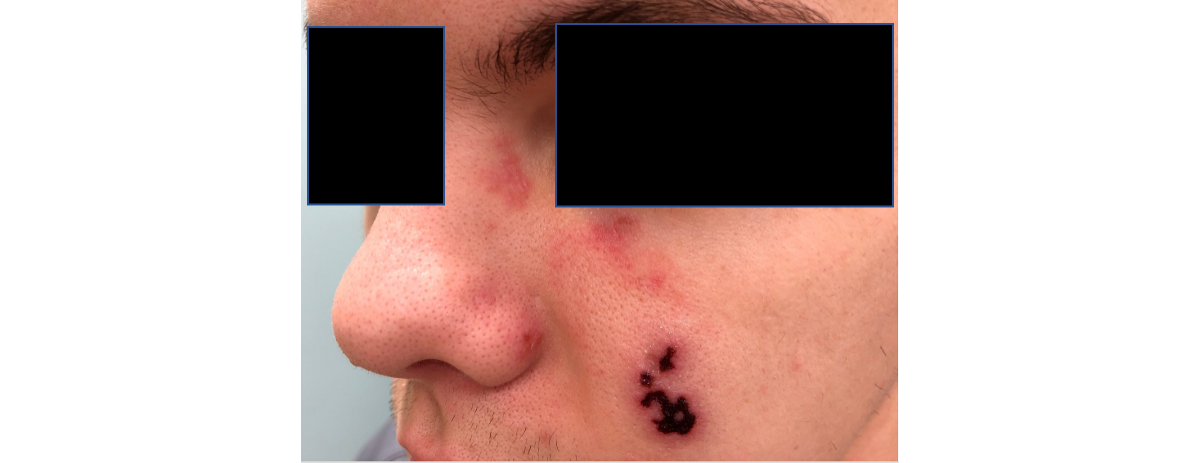
Case 3. Images from clinic 1 week later.

A 26-year-old male with a 3-day history of a round pink plaque on the left cheek, within which were papules and erosions, was referred to teledermatology. At the time of his referral, the referring provider had prescribed treatment of this plaque with topical clotrimazole. The patient reported that he had worn a mask and participated in paintball and jiujitsu a few days prior to presentation and that his lesions appeared shortly afterwards. He reported that the lesion started as a pimple or vesicle, and then progressed into a plaque. A single submitted clinical image showed a limited view of the left cheek (Figure 6). The teledermatologist’s differential diagnosis included impetigo, tinea faciei, Majocchi granuloma, and contact dermatitis. The teledermatologist advised the referring provider to obtain a superficial bacterial culture of the plaque, continue clotrimazole, and start empiric treatment with doxycycline if the patient was unable to return for the culture. The patient subsequently reported progression of his rash and was scheduled for an in-person visit with the dermatologist 1 week later. At that time, the initial lesions had crusted over, and new lesions on his left upper medial cheek, left nasal bridge, and left nasal ala in a dermatomal distribution were noted (Figure 7). A clinical diagnosis of herpes zoster was made, the patient was prescribed oral acyclovir, and the rash subsequently resolved.

### Case 4

**Figure 8:**
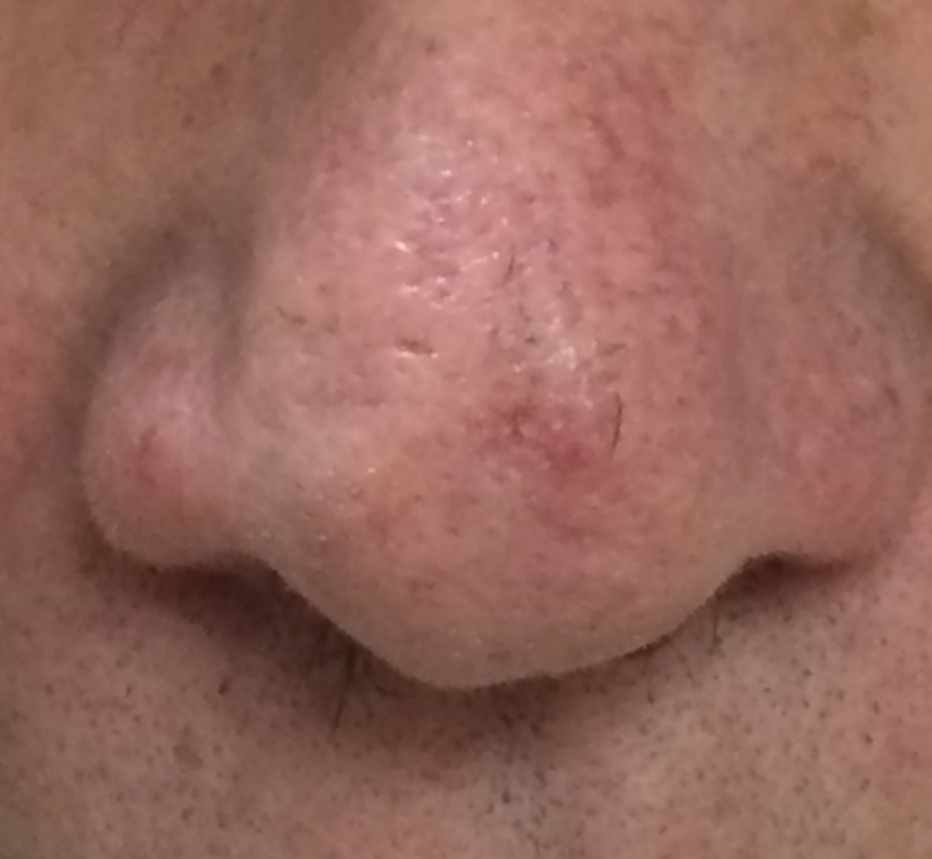
Case 4. Submitted image to teledermatology showing erythematous macules with apparent scale or crust on the nasal tip and ala.

A 63-year-old man was referred to teledermatology for evaluation of a 7-month history of an enlarging nasal lesion. The teledermatologist reviewed the image and described erythematous macules that appeared to have scale or crust on the nasal tip and ala (Figure 8). The differential diagnosis provided by the teledermatologist was dependent on textural characteristics and included actinic keratoses if the lesion was rough and excoriated papulopustular rosacea if the texture was not rough. The teledermatologist requested additional textural information. Due to the incomplete information on skin texture, the patient was referred for an in-person visit, where his exam revealed no overlying scale or roughness to suggest actinic keratosis and no features suggestive of squamous cell carcinoma. He denied a history of facial flushing or acneiform or pustular eruptions. He was clinically diagnosed with a telangiectasia, likely due to dermatoheliosis, and no further treatment was recommended.

### Case 5

**Figure 9:**
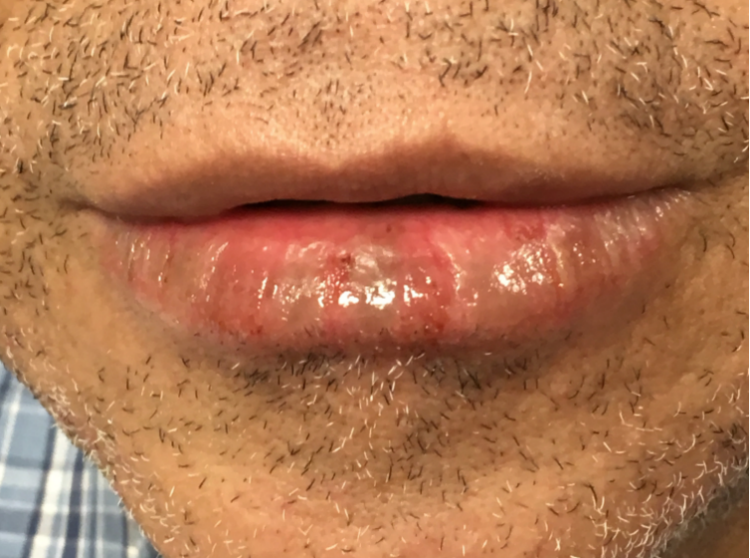
Case 5. Submitted teledermatology image of focal erosion with hemorrhagic crust and vesiculation on lips.

**Figure 10:**
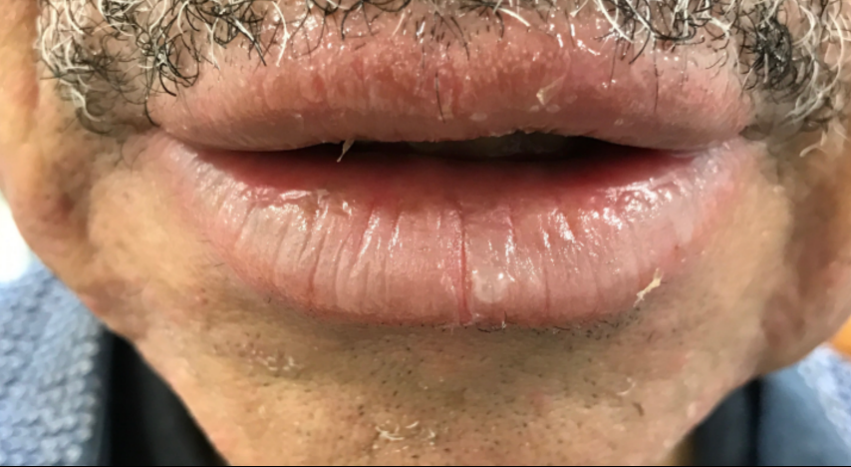
Case 5. Image from first in-person visit showing resolving vesicles and erosions.

**Figure 11:**
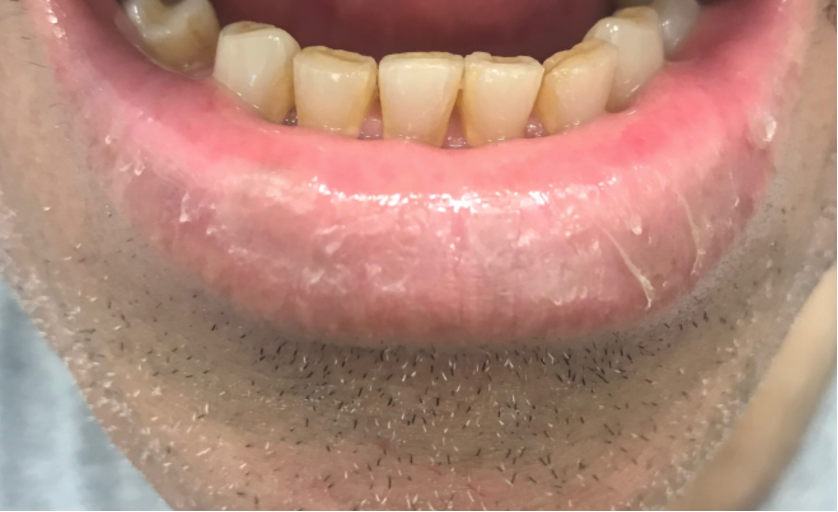
Case 5. Image from second in-person visit, when biopsy was taken.

A 69-year-old man with a 2-month history of blistering lips with skin peeling and pain unresponsive to Vaseline was referred to teledermatology. The submitted image showed a focal erosion with hemorrhagic crust and vesiculation (Figure 9), and the differential included herpes simplex virus (HSV), erythema multiforme, contact dermatitis, pemphigus vulgaris, and paraneoplastic pemphigus. The teledermatologist pointed out that the patient was not asked about history of similar eruptions, involvement of the oral mucosa, or associated symptoms including pain or burning, which would have aided the diagnosis. The consultant also recommended obtaining HSV/varicella zoster virus viral cultures and applying emollient, and the patient was scheduled for an in-office dermatology appointment. During the first in-person visit, the erosions and vesicles were resolving (Figure 10), and a bacterial culture was taken from a focal erosion which grew methicillin-resistant *Staphylococcus aureus*. He was treated with doxycycline and the fissure healed. The lip erosions subsequently recurred (Figure 11) and were biopsied, with pathology most consistent with a lichenoid dermatitis. He was treated with triamcinolone 0.1% cream and his symptoms resolved.

### Case 6

**Figure 12:**
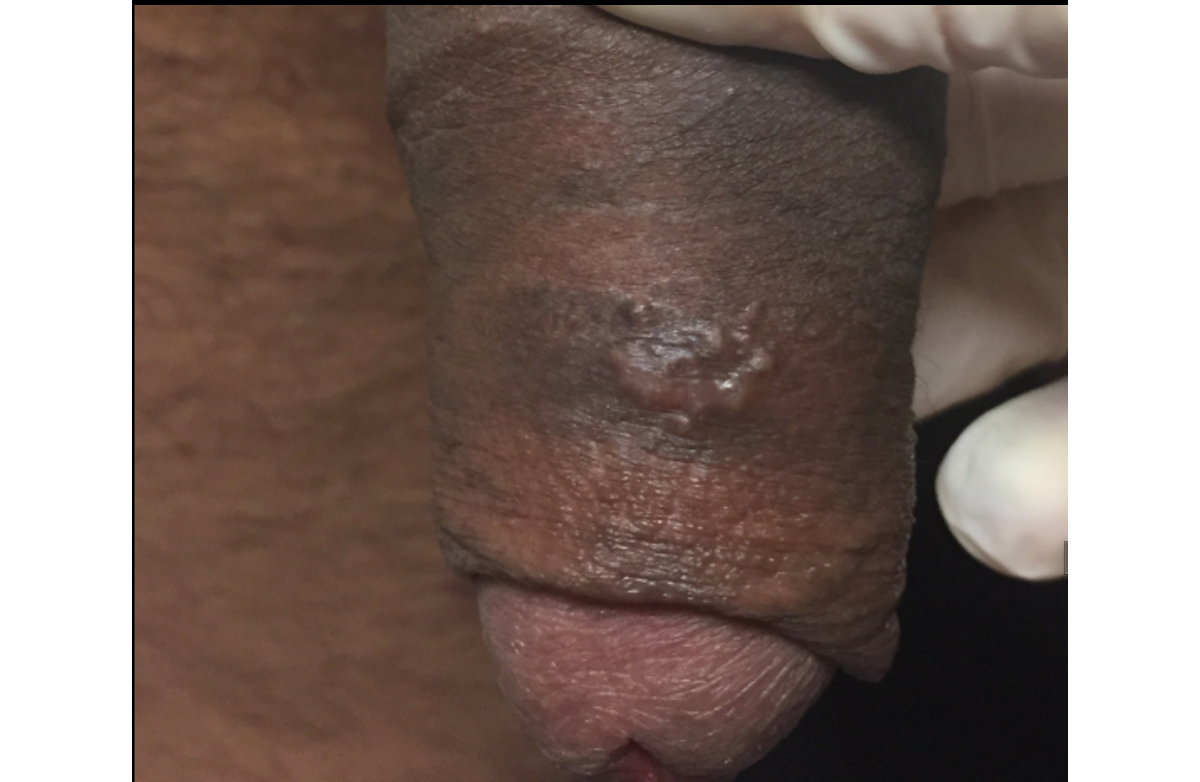
Case 6. Submitted teledermatology image suggestive of vesicles versus pustules on the dorsal penile shaft.

**Figure 13:**
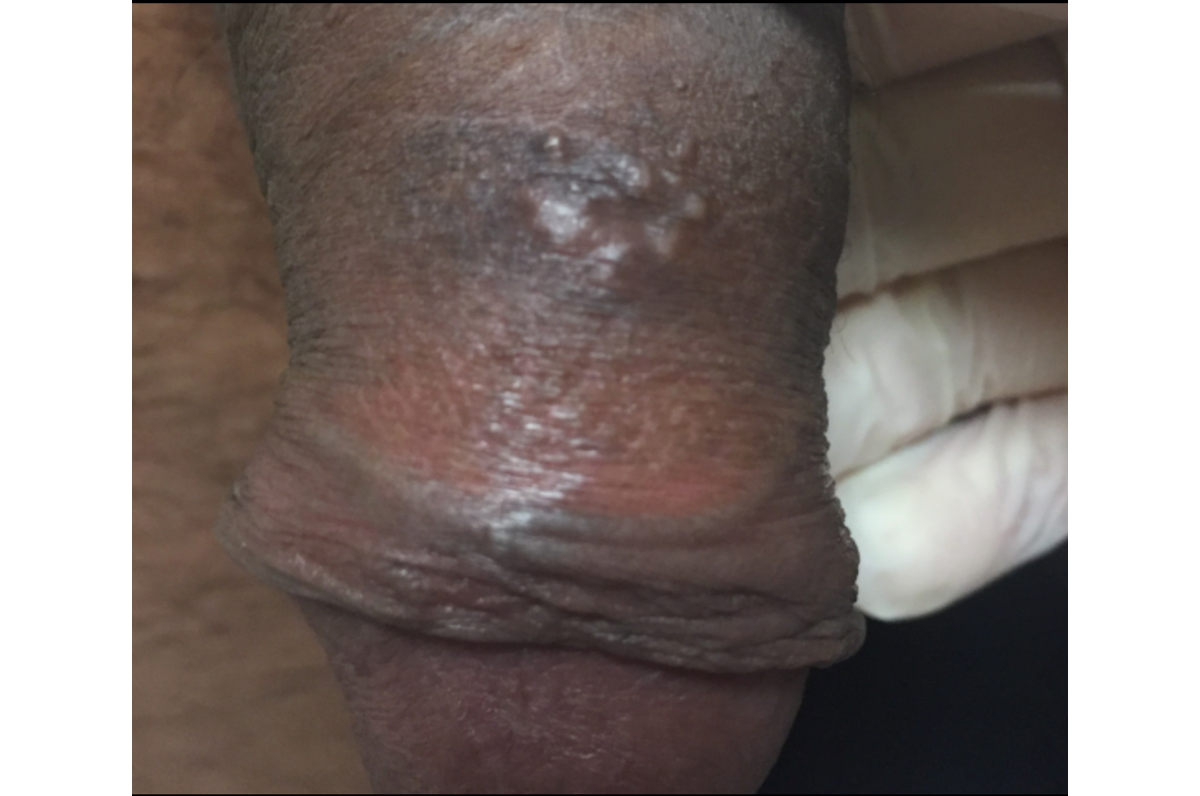
Case 6. Image from in-person visit.

A 41-year-old man with no known history of sexually transmitted infections was referred to teledermatology for 1 month of an unchanging nontender penile rash. He was in a monogamous relationship, and his female partner did not have a similar rash. Submitted images demonstrated a cluster of apparent deep-seated vesicles or pustules on the dorsal penile shaft (Figure 12) as well as documented 1.5-2 cm suprapubic lymphadenopathy. The teledermatologist noted difficulty in distinguishing between vesicles and pustules in the images and recommended obtaining a medication history as well as viral and bacterial swab culture for genital vesicles and pustules. The differential included infectious and inflammatory etiologies, including HSV, lymphogranuloma venereum, and a fixed drug eruption. The patient was scheduled for an in-person evaluation (Figure 13), during which a shave biopsy was obtained that demonstrated a foreign body giant cell reaction suggestive of a ruptured epidermal inclusion cyst.

### Case 7

**Figure 14:**
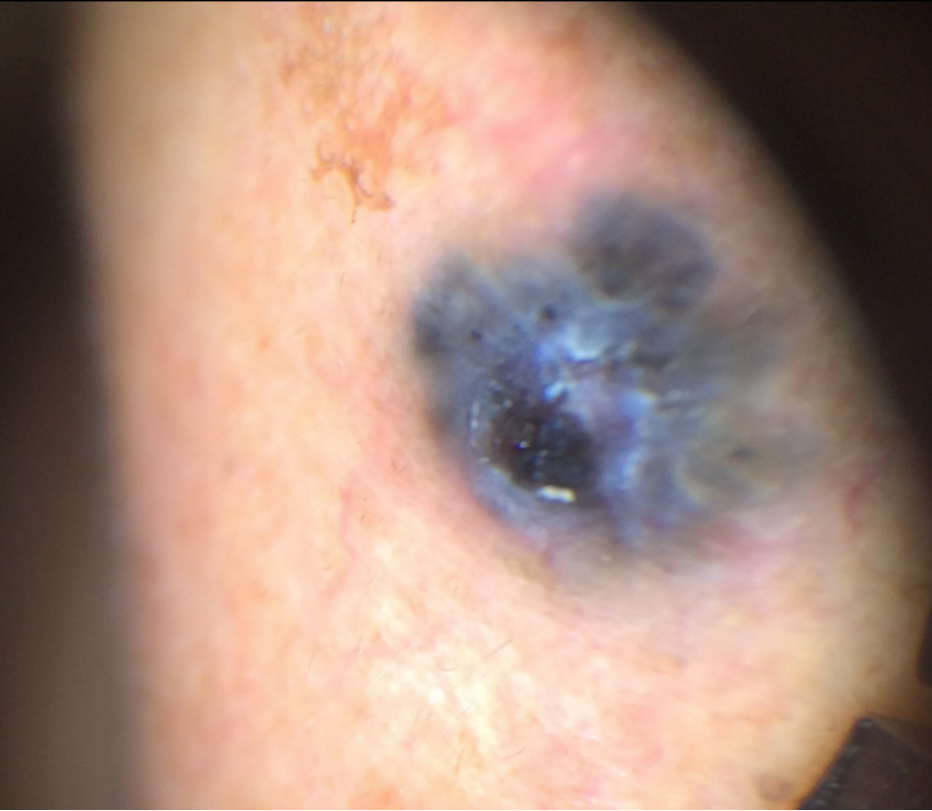
Case 7. Submitted image to teledermatologist taken with dermoscopy.

**Figure 15:**
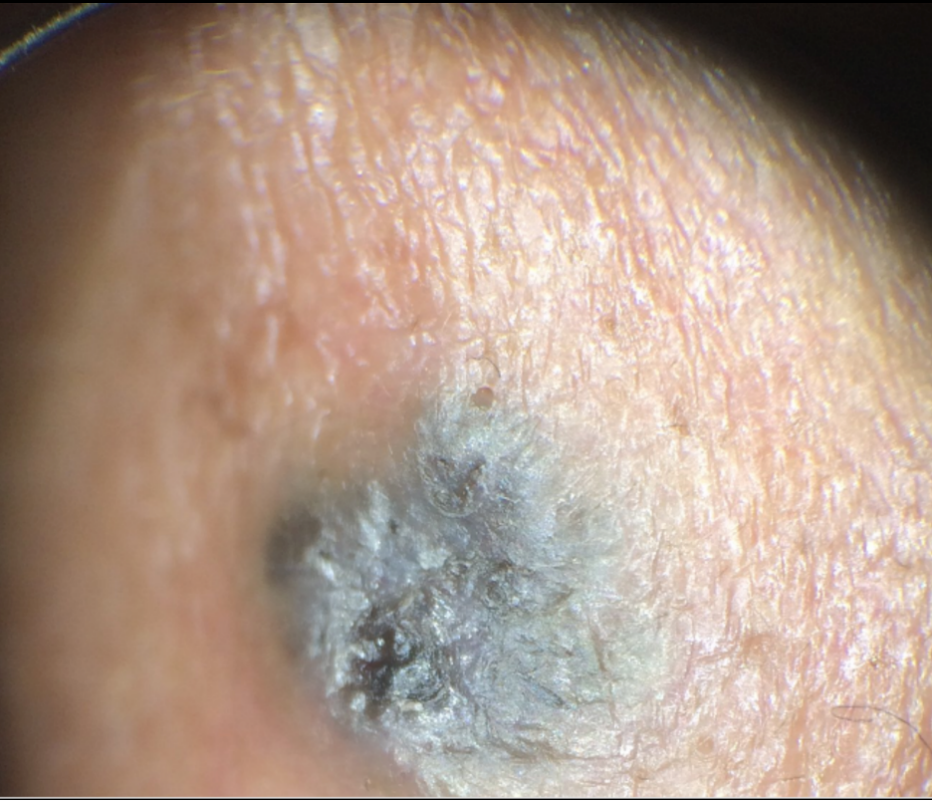
Case 7. Submitted image to teledermatologist taken with dermoscopy.

**Figure 16:**
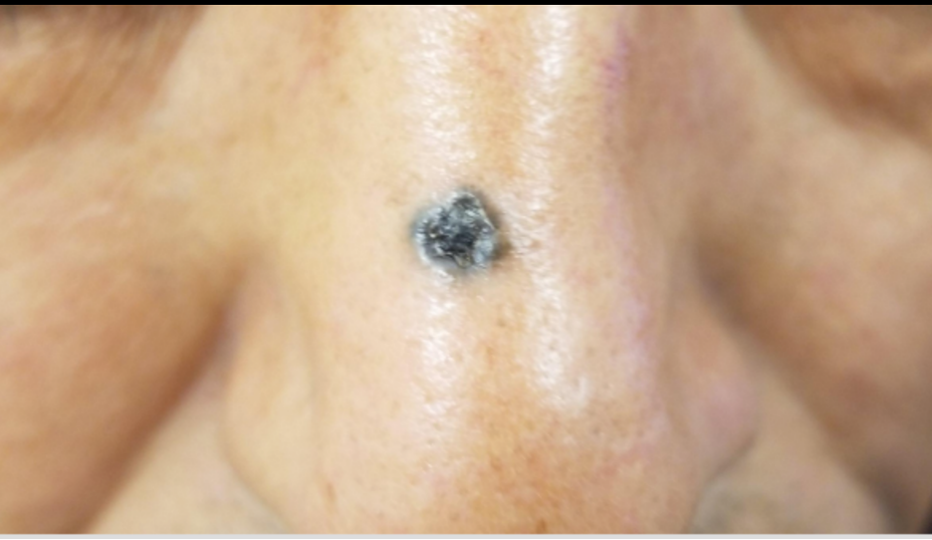
Case 7. In-person image showing 5-6 mm black thin papule with collarette of scale on the nasal bridge.

A 66-year-old man with a 1- to 2-year history of a hyperpigmented nasal papule was referred for teledermatology consultation. The teledermatologist noted that the two dermatoscopic images provided were poorly focused (Figures 14 and 15), and no gross images were submitted. Thus, the consultant was unable to provide a differential diagnosis with the provided clinical images, and the patient was referred for an in-person visit. During the in-office encounter, exam revealed a 5-6 mm black thin papule with a collarette of scale on the nasal bridge (Figure 16) with a differential of irritated seborrheic keratosis versus melanoma. A shave biopsy was performed of the lesion, which resulted in a diagnosis of pigmented basal cell carcinoma. The patient was referred for Mohs surgery.

## Discussion

### Principal Results

From our case analysis, we identified multiple factors that likely contributed to diagnostic discordance between SAF teledermatology consultations and in-person visits in these cases. One contributing factor was poor image quality, including use of bright lighting creating shine artifact (case 1), submission of photos that showed partial views without showing the entire anatomic area involved (cases 1, 2, 3, and 7), and poorly focused images (case 7). These cases demonstrate the importance of education on appropriate image acquisition techniques. Following previously published best practices [21,22] and ongoing feedback to providers submitting photos may facilitate improved photo quality over time.

In some cases, additional clinical history or diagnostic tests provided by the referring clinician would have been helpful in narrowing the differential diagnosis. For example, diagnostic accuracy may have increased if the referring provider had performed bedside diagnostics such as superficial wound cultures, viral cultures, KOH preps (cases 2, 3, and 5), or dermoscopy (case 7). Use of dermoscopy has previously been shown to be a helpful tool in teledermatology programs, particularly in the evaluation of pigmented lesions [12]. Improved training of referring providers in using these diagnostic modalities may be helpful by providing clinical data to the dermatologist that leads to improved diagnostic accuracy. In addition, many cases illustrate the importance of a complete relevant medical history. For example, in case 6, a clinical history of similar vesicular eruptions, involvement of the oral mucosa, new exposures in the affected area, and associated pain or burning would have been helpful in differentiating between HSV, erythema multiforme, contact dermatitis, or pemphigus. Similarly, increased education for referring providers around questions relevant to certain dermatologic presentations (ie, asking about involvement of oral mucosa for bullous eruptions) would help them obtain an optimal history to aid in diagnosis.

Even with high-quality images, some morphologic characteristics may be difficult to appreciate with photos given visual limitations and inability to evaluate textural characteristics. For example, in case 4, palpation for detection of scale and induration may have helped the teledermatologist differentiate actinic damage from telangiectasias, and the teledermatologist interpreted the initial submitted image as a lesion with apparent overlying scale or crust, which was not seen in person. Case 6 also highlights that cystic, pustular, and vesicular structures can sometimes be difficult to distinguish from photos alone, depending on the angle and lighting of the photo taken. These cases highlight inherent diagnostic limitations of teledermatology services.

Finally, some factors such as atypical presentations and evolution of skin lesions over time were not specific to teledermatology and may have occurred in an initial in-person visit as well. For example, multiple cases remained diagnostically challenging even when patients were seen in person due to atypical presentations (cases 1, 5, and 6). Additionally, cases 2 and 3 highlight how morphology and distribution can evolve over time, leading to changes in suspected diagnosis. Teledermatology may have the highest utility for cases with typical presentations as unusual presentations may be difficult for teledermatologists to manage confidently without in-person evaluation and possible skin biopsy. Recognition of these limitations may also help with appropriate selection of patients more likely to benefit from an in-person encounter rather than a teledermatology visit. Even when patients can be managed with teledermatology, it is important for patients and providers to maintain follow-up to ensure appropriate response to management and ongoing support if the patient’s condition or morphology changes from the time of original teledermatology consultation.

Our study has important implications given that the use of asynchronous and other types of virtual care continues to rise [9,10], as incorrect diagnosis via teledermatology or lack of a timely referral for an in-person visit may have potential negative consequences for patients. For example, case 2 highlights how a teledermatologist’s incorrect diagnosis and prescription of betamethasone for tinea corporis may have contributed to the progression of the rash, although the correct diagnosis also may not have been made at an initial in-person visit given its atypical presentation. In case 7, it was essential that a timely referral was made given the indeterminate images, which allowed the patient to receive a biopsy resulting in a diagnosis of pigmented basal cell carcinoma.

In order to improve the quality of SAF consultations and decrease rates of diagnostic discordance, we advocate for use of a standardized guide to help improve the quality of SAF teledermatology consults, including image quality and appropriate workup. In addition to guidelines already outlined by the American Telemedicine Association [21,22], our study highlights the need for guidelines for proper lighting, examples of dermoscopy images, relevant questions to ask in the patient history for certain morphologic presentations, and certain suggested diagnostic tests to perform before submitting a consult.

In addition, the educational value of SAF consults should continue to be emphasized to both referring and consulting providers in order to help improve the quality of consults and ensure the highest level of diagnostic accuracy. Referring providers’ ability to obtain an optimal history and diagnostic testing will also likely improve with increased use of SAF teledermatology and iterative dialogue between providers about patients’ medical management. For example, previous studies have highlighted the educational potential of SAF teledermatology systems on improving referring primary care provider knowledge of dermatologic care [23]. Teledermatologists should also be encouraged to engage in education with referring providers to ensure this ongoing learning process.

The strengths of our study include its in-depth analysis of specific cases and side-by-side comparison of teledermatology and in-person consults for the same patients. While several studies have been published on overall rates of diagnostic discordance, which have been estimated at around 39%-67% [12-17,19], none to our knowledge have presented a case-by-case analysis that illustrates and compares the teledermatology and in-person visits for the same patients.

### Limitations

Some of the limitations of our study include that our sample size limits our ability to generalize across all cases of diagnostic concordance, although we intend for this to be a more in-depth study of fewer cases. In addition, only one teledermatologist reviewed each image submitted by the referring provider, which may introduce the possibility that diagnostic uncertainty may have been due to the individual teledermatologist’s level of comfort with the diagnosis rather than the inherent limitations of teledermatology. The teledermatologist was also different than the in-person dermatologist in all but the second case, and thus some of the differences in experience and comfort level between the teledermatologists and in-person dermatologists may have contributed to the discordant diagnoses.

### Conclusions

Collectively, these cases highlight factors that can contribute to diagnostic discordance between teledermatologist and in-person dermatologist and the importance of ensuring that teledermatology services are supported by readily available in-person visits when appropriate to achieve the correct diagnosis in these cases. We also highlight the importance of ongoing education of referring providers to ensure optimal history and diagnostic workup and improve quality of consultations and the development of standardized guidelines for submitting referrals.

Teledermatology can provide substantial benefits to patients, and recognition of its limitations and mitigation of the factors identified in these cases provide opportunities to improve the quality and diagnostic accuracy of SAF teledermatology services.

## Abbreviations

HSV: herpes simplex virus
KOH prep: potassium hydroxide preparation
SAF: store-and-forward
